# Evolution of aortic wall thickness: long-term follow up from the Multi-Ethnic Study of Atherosclerosis (MESA)

**DOI:** 10.1186/1532-429X-15-S1-M14

**Published:** 2013-01-30

**Authors:** Chia-Ying Liu, Doris Chen, Gisela Teixido-Tura, Colin O Wu, Atul R Chugh, David A Bluemke, Joao A Lima, W Gregory Hundley

**Affiliations:** 1 Department of Radiology, Johns Hopkins University, Baltimore, MD, USA; 2 Office of Biostatistics Research, National Institutes of Health, Bethesda, MD, USA; 3 Department of Cardiology, Hospital General Universitari Vall dHebron, Barcelona, Spain; 4 Division of cardiovascular medicine, University of Louisville School of Medicine, Louisville, KY, USA; 5 Radiology and Imaging Sciences, National Institutes of Health, Bethesda, MD, USA; 6 Division of Cardiology, Deparment of Internal Medicine, Wake Forest University, Winston-Salem, NC, USA

## Background

A number of studies have been published over the years concerning the relationship between the thickened intima of human arteries and atherogenesis. Increased arterial wall thickness is associated with the prevalence and incidence of cardiovascular disease. Age-related increases of aortic wall thickness have been reported in several cross-sectional community-based studies. However, longitudinal changes of these measurements have not yet been documented. The purpose of this study is to characterize age- and sex-specific aortic wall thickness (AWT) distributions and yearly rates of change in older adults.

## Methods

371 longitudinal and 426 cross sectional studies with AWT images by MRI were analyzed. MRI was performed at first in MESA1 (baseline, 2000-2001), and then in MESA5 (ten-year follow-up, 2010-2011). Both exams used 1.5-T whole-body MRI systems. Images were obtained using a double inversion recovery black-blood fast spin-echo sequence with ECG gating. Axial images of the descending thoracic aorta were obtained at the level of the right pulmonary artery. The thickness of the midthoracic descending aortic wall was measured using electronic calipers at 4 standard positions: 12, 3, 6, and 9 o'clock (QMASS 7.2). The average value of these 4 measurements was calculated.

## Results

**Figure 1:**
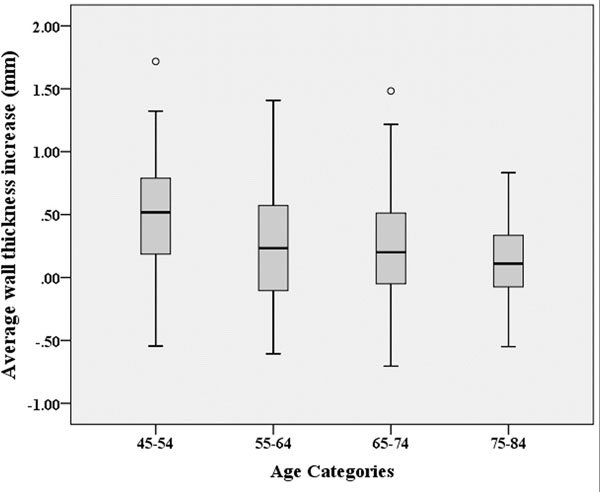
Average aortic wall thickness increase in ten years by age categories (in the baseline age).

Table 1 lists demographics and AWT in MESA5 (2010-2011), and changes of AWT in ten years stratified by gender. AWT and cardiac function were significantly different between men and women. Changes of AWT were greater for men than women, but not significant. The average yearly rate of AWT change was 0.032mm. Figure 1 displays the average AWT increase in ten years by age categories (in the baseline age). AWT increased more markedly in mid-adulthood, and was plateaued over time into late-adulthood (p-trend < 0.001). Framingham global CVD risk score assessed at the baseline was positively correlated with the AWT measured in MESA5 (R=0.261, p < 0.001).

**Table 1:** Mean characteristics of the MESA5 participants

At MESA5 (2010-2011), mean±SD	GLOBAL (N=426)	Women (N=245)	Men (N=181)	p-value
Age (years)	70.8±8.7	70.9±8.9	70.5±8.5	0.64
Race (N of white/black)	259/167	144/101	115/66	0.27
Body mass index (kg/m2)	28.7±5.3	28.7±5.9	28.3±5.3	0.45
Systolic blood pressure (mmHg)	125±21	125±21	124±21	0.68
Diastolic blood pressure (mmHg)	68±10	65±10	71±10	< 0.001
Metabolic Syndrome, N (%)*	146(34.3)	96(39.2)	50(27.6)	0.02
LV End diastolic volume (ml)	120±31	106.2±23	138.4±31	< 0.001
LV End systolic volume (ml)	47±18	38.5±12	57.5±20	< 0.001
LV End diastolic mass (g)	125±35	105.4±22	152.2±32	< 0.001
LV Stroke volume (ml)	73.2±18	67.7±16	80.9±18	< 0.001
LV Ejection Fraction (%)	61.8±7.2	63.9±6.7	58.9±7	< 0.001
Aortic wall thickness (mm)	2.67±0.27	2.61±0.26	2.75±0.26	< 0.001
Wall thickness difference in ten years (mm)	0.32±0.46	0.30±0.44	0.35±0.48	0.26

* Metabolic Syndrome was defined by NCEP guidelines (Circ 2004;109;433-438)

## Conclusions

We report aortic wall thickness changes over mid to late adulthood in longitudinal comparisons. Further analyses will reveal the correlates of these alterations with clinical variables.

## Funding

N01-HC-95168 from the National Heart, Lung, and Blood Institute

